# LPS-induced monocarboxylate transporter-1 inhibition facilitates lactate accumulation triggering epithelial-mesenchymal transformation and pulmonary fibrosis

**DOI:** 10.1007/s00018-024-05242-y

**Published:** 2024-05-06

**Authors:** Jinhua Feng, Han Zhong, Shuya Mei, Ri Tang, Yang Zhou, Shunpeng Xing, Yuan Gao, Qiaoyi Xu, Zhengyu He

**Affiliations:** 1 Department of Critical Care Medicine, Ren Ji Hospital, Shanghai Jiao Tong University School of Medicine, Shanghai, 200127 China; 2 Key Laboratory of Anesthesiology (Shanghai Jiao Tong University), Ministry of Education, Shanghai, 200127 China

**Keywords:** Lipopolysaccharide, Lactate, Epithelial-mesenchymal transformation, Monocarboxylate transporter-1, Pulmonary fibrosis

## Abstract

The epithelial-mesenchymal transformation (EMT) process of alveolar epithelial cells is recognized as involved in the development of pulmonary fibrosis. Recent evidence has shown that lipopolysaccharide (LPS)-induced aerobic glycolysis of lung tissue and elevated lactate concentration are associated with the pathogenesis of sepsis-associated pulmonary fibrosis. However, it is uncertain whether LPS promotes the development of sepsis-associated pulmonary fibrosis by promoting lactate accumulation in lung tissue, thereby initiating EMT process. We hypothesized that monocarboxylate transporter-1 (MCT1), as the main protein for lactate transport, may be crucial in the pathogenic process of sepsis-associated pulmonary fibrosis. We found that high concentrations of lactate induced EMT while moderate concentrations did not. Besides, we demonstrated that MCT1 inhibition enhanced EMT process in MLE-12 cells, while MCT1 upregulation could reverse lactate-induced EMT. LPS could promote EMT in MLE-12 cells through MCT1 inhibition and lactate accumulation, while this could be alleviated by upregulating the expression of MCT1. In addition, the overexpression of MCT1 prevented LPS-induced EMT and pulmonary fibrosis in vivo. Altogether, this study revealed that LPS could inhibit the expression of MCT1 in mouse alveolar epithelial cells and cause lactate transport disorder, which leads to lactate accumulation, and ultimately promotes the process of EMT and lung fibrosis.

**Supplementary Information:**

The online version contains supplementary material available at 10.1007/s00018-024-05242-y.

## Introduction

Sepsis is characterized by an dysregulated host response to infection accompanied by acute organ dysfunction and a high risk of death [1]. Although numerous studies have improved the early identification and treatment of sepsis in the past few years, acute respiratory distress syndrome (ARDS) caused by sepsis and the concomitant lung fibrosis led to poor outcomes in patients [2, 3]. Therefore, an in-depth study of the pathogenesis of sepsis-associated pulmonary fibrosis, timely blocking early stage of sepsis-associated pulmonary fibrosis, and improving the prognosis of patients are urgent problems to be solved in the clinical practice of intensive care medicine.

The epithelial-mesenchymal transformation (EMT) refers to the biological process of epithelial-to-mesenchymal transition, which plays an important role in embryonic development, chronic inflammation, tissue remodeling, cancer metastasis, and a variety of fibrotic diseases [4–6]. There is growing evidence that EMT process in alveolar epithelial cells is closely associated with the pathogenesis of pulmonary fibrosis [4, 7–9]. Kottmann's study in 2012 showed that lactate could reduce the pH value of extracellular fluid and promote the activation of latent Transforming growth factor-β1 (TGF-β1) to TGF-β1, which could initiate the EMT process and finally progress to pulmonary fibrosis [10, 11]. Our previous studies have found lactate accumulation in lung tissue of mice model of sepsis-associated pulmonary fibrosis [12, 13]. However, it is uncertain how LPS facilitates the development of sepsis-associated pulmonary fibrosis by promoting lactate accumulation in lung tissue and thus initiating EMT process. Under normal circumstances, the lactate produced by cell metabolism could be transported into the cells by monocarboxylic acid transporter-1 (MCT1), thereby reducing the concentration of lactate in the extracellular fluid [14]. LPS has been shown to inhibit MCT1 expression [15]. Therefore, we hypothesized that LPS inhibition of MCT1 expression may play an important role in lactate accumulation and EMT during the process of sepsis-associated pulmonary fibrosis.

In this study, in vitro and in vivo experiments have been engaged to explore the connections among MCT1 modulation, extracellular lactate accumulation, EMT and pulmonary fibrosis. Our study proved that LPS could inhibit the expression of MCT1 in mouse alveolar epithelial cells and cause lactate transport disorder, which leads to lactate accumulation in extracellular fluid, and ultimately promotes the process of EMT and tissue fibrosis. Our findings may afford opportunities to seek promising therapeutic targets of sepsis-associated pulmonary fibrosis.

## Materials and methods

### Animal grouping and treatments

A total of 48 healthy mice were randomly divided into sham (n = 6), LPS (n = 18), MCT1OE (n = 6), MCT1OE + LPS (n = 18) groups. In LPS group and MCT1OE + LPS group, animals were injected intraperitoneally with LPS (5 mg/kg, *Escherichia coli* O127:B8, Sigma, USA) for 3 consecutive days while mice were injected with saline instead in sham group and MCT1OE group, which was optimized on the basis of other investigator and our previous research [13, 16, 17].

In order to establish an alveolar epithelial cells specific MCT1-overexpressing (MCT1OE) mouse model, intratracheal administration of 40 μl MCT1OE adeno-associated virus (AAV) mixture (AAV concentration: 5 × 10^12^ VG/ml), purchased from Genomeditech (Shanghai, China), was delivered to each mouse 4 weeks before LPS (saline) injection. The mice were anesthetized and sacrificed 7 days after the administration of LPS or saline, with the serum, lungs and bronchoalveolar lavage fluid (BALF) samples collected for the following investigations.

All animals were supported by Shanghai SLAC Laboratory Animal, China. Male C57BL/6 mice aged 7–8 weeks (20–25 g) were housed under diurnal lighting status with food and water ad libitum.

### Cell culture

Mouse alveolar epithelial cells (MLE-12) were obtained from FuHeng Cell Center (Shanghai, China). The cells were incubated in medium containing 10% fetal bovine serum (FBS, Gibco, USA), 100 IU/mL penicillin, 100 IU/mL Streptomycin, insulin 0.005 g/L, transferrin 0.01 g/L, selenite sodium 30 nmol/L, hydrocortisone 10 nmol/L, β-estradiol 10 nmol/L, HEPES 10 mmol/L and L-glutamine 2 mmol/Lin a humidified incubator with 5% CO_2_ atmosphere at 37 °C. Culture media were replaced every day. After reaching 80%–90% confluence, MLE-12 cells were seeded into 6-well plates at a density of 2 × 10^5^ cells/mL (2 ml in each well), and stimulated with 1 μg/mL LPS or phosphate buffer saline (PBS) as control.

### MCT1OE cell lines

We subcloned a coding sequence (Gene ID: 20501) of MCT1 into PGMLV-6395 vector to generate lentiviral for overexpression of MCT1. Then human embryonic kidney 293 T (HEK 293 T) cells were co-transfected with packaging mix including MCT1OE lentivirus or negative control vector. These cells were cultured for 48 h. After the incubation, the packaged lentiviruses including MCT1OE lentivirus or negative control vector (NC group) were collected and used to infect MLE-12 cells respectively. After 2 days, stable-transfected cells were selected with 10 µg/mL of puromycin (Genomeditech, China).

### Knock down of MCT1

Mouse MCT1 small interfering RNA (siRNA) was purchased from Genomeditech (Shanghai, China). The primer sequences of MCT1 siRNA were: 5′-CCAGUGAAGUAUCAUGGAUAU-3′ and 5′-AUAUCCAUGAUACUUCACUGG-3′. A negative control siRNA was transfected into the cell line as a negative control (NC group). All transfections were carried out according to the manufacturer’s instructions using Lipofectamine 3000 (beyotime, China).

### Histopathology and immunofluorescence analysis

The lung tissues were fixed in 4% formaldehyde, embedded in paraffin, sliced and dewaxed. Afterwards, the lung sections were stained with hematoxylin and eosin (H&E) and as well as Masson’s trichrome following standard protocols.

In addition, antigens of lung sections were recovered with citric acid buffers. After exposure to 10% bovine serum albumin (BSA) for one hour, the sections were incubated with primary antibodies against Vimentin (A19607, Abclonal, China), E-cadherin (14472S, CST, USA), and MCT1 (A3013, Abclonal, China) overnight at 4 ℃. After triple washing, sections were incubated with fluorescein-conjugated antibody for 1 h. Finally, the images were captured by fluorescence microscope (Leica, DMi8, Germany) after nucleus staining with 4′6-diamino-2-phenylindole (DAPI, Southern Biotech, USA). Fiji software (2.15.1) was used to quantify the immunofluorescence in the result. The mean gray value (fluorescence intensity/area) of each immunofluorescence image was detected and analyzed [18].

### Cell immunofluorescence

MLE cells were seeded on the coverslip, and then were fixed with 4% paraformaldehyde at room temperature (RT) for 15 min. After incubation with blocking buffer (10% BSA) at RT for 30 min, cells were then permeabilized in 0.1% Triton X-100 for 15 min at RT. Then cells were incubated with primary antibodies diluted in blocking buffer overnight at 4 °C in the humid chamber. After washing for three times in PBS, cells were then stained with secondary antibodies conjugated with fluorescence. Then the cells were washed in PBS with three times again before mounting with DAPI (Southern Biotech, USA). The primary antibodies used in the study was anti-Vimentin (A19607, Abclonal, China). Images were captured using the confocal microscope (Olympus Fluoview FV3000). As mentioned above, each immunofluorescence image was detected and analyzed by Fiji software.

### Western blotting

Western blot was used to detect the protein expression levels of MCT1, E-cadherin (E-cad), Vimentin, collagen-I α-1 (COL1A1) and α-smooth muscle actin (α-SMA). Protein lysates were extracted from lung tissue or cells using radioimmunoprecipitation assay buffer with 1% protease inhibitor cocktail, and 1% phosphatase inhibitor cocktail (Epizyme Biomedical Technology, China). After centrifugation, the supernatants of lysates were collected for following assays. The protein concentration of the supernatants was measured by a BCA protein assay kit (Epizyme Biomedical Technology, China). Equal weight of protein mixture was separated by 4%–15% SDS-polyacrylamide gel electrophoresis, and transferred to polyvinylidene difluoride membranes (Millipore, USA). Then the membranes were blocked with 3% BSA for one hour. After that the membranes were incubated with appropriate primary antibodies (MCT1 (A3013, ABclonal, China), E-cadherin (14472S, CST, USA), COL1A1 (72026S, CST, USA), α-SMA (19245, CST, USA), Vimentin and β-Actin (8457S, CST, USA)) and secondary antibodies respectively. The dilution of antibodies was 1:1000 to 1:2000. The chemiluminescence blots were visualized by Image Lab software (Bio-Rad, USA) with Enhanced ECL Kit (Vazyme, China).

### Lactate level assay

The levels of lactate in supernatants of MLE-12 cell medium were measured with a lactate assay kit (Nanjing Jiancheng Bioengineering, China). The cell supernatant was thawed on ice after being stored at ultra-low temperature. The working solution was added to the centrifuge tube according to the instructions, followed by the addition of the test samples and the blank samples. After sample addition was completed, the centrifuge tubes were placed in a 37 °C water bath and the termination solution was added after 10 min. Subsequently, 250 µl of the solution were transferred sequentially to a 96-well plate with a pipette, and the absorbance of the solution was measured at a wavelength of 530 nm using a microplate reader (Berthold, LB942s, Germany). Sample concentrations were calculated by the following formula (A = (a−b)/ (c−b) × C × M, A = Lactate concentration, a = OD value of the test samples, b = OD value of the blank samples, c = OD value of the standard samples, C = 3 mmol/L, M = multiple of dilution). Meanwhile, the levels of lactate in BALF of mice were detected with mouse lactate ELISA kit (Lenton, China). BALF was collected by insufflation of 500 µL of cold phosphate-buffered saline into the alveoli of mice through an endotracheal tube. The supernatant was obtained by centrifugation at 3000 g for 10 min and then stored in an ultra-low temperature refrigerator. The experiment procedure followed the manufacturer’s instructions.

### Statistical analysis

All experiments were repeated three times at least. The continuous data were presented as mean ± SD and analyzed using GraphPad Prism 8.0 software (USA). One-way analysis of variance (ANOVA) was conducted for multiple comparisons. Student’s t-test (two tailed) was used to compare the difference between two groups. A p-value of < 0.05 was considered as a significant difference.

## Results

### Abundant exogenous lactate stimulated MCT1 expression and EMT

**Fig. 1:**
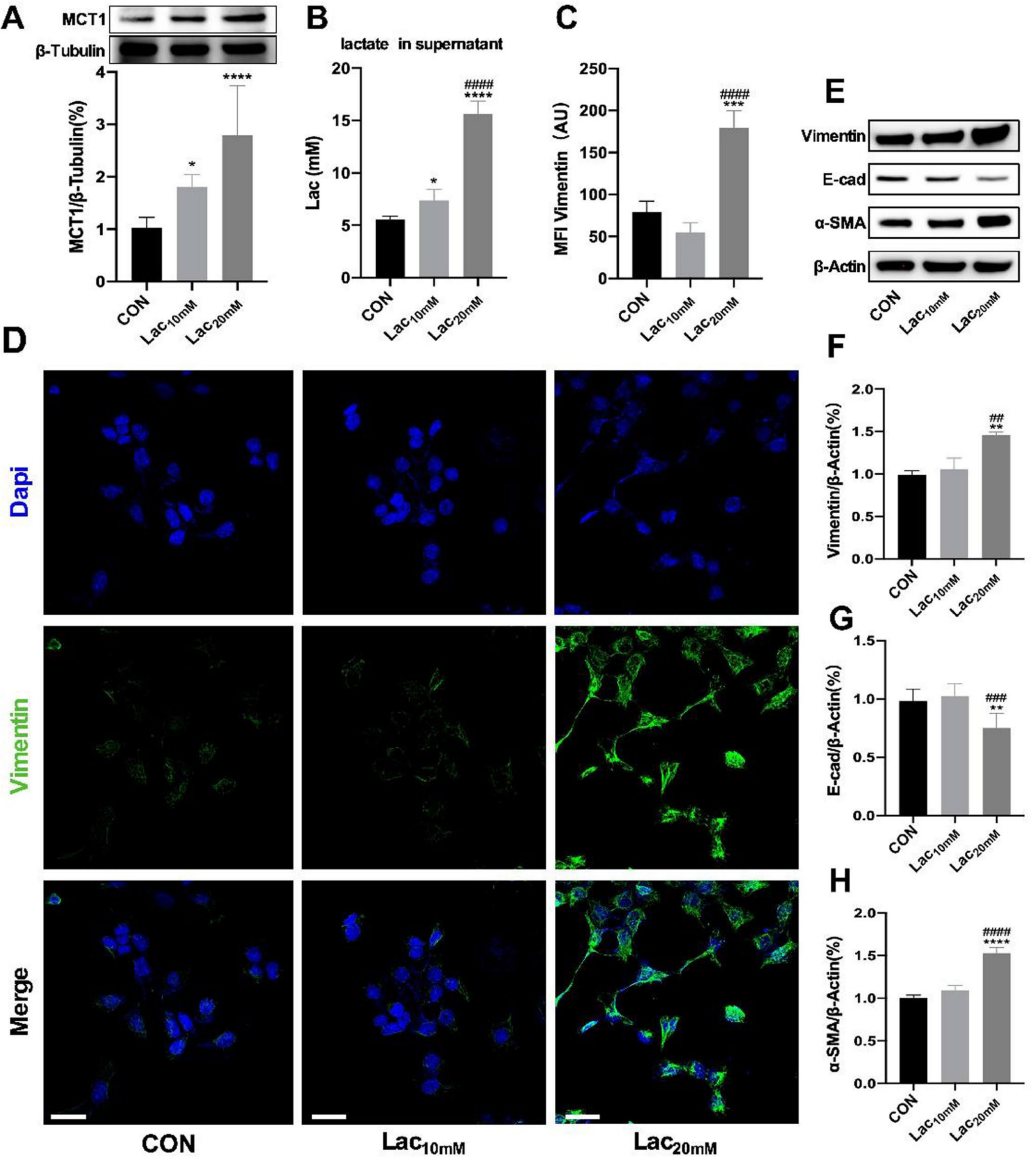
Abundant exogenous lactate stimulated MCT1 expression and EMT. MLE-12 was treated with 10 mM or 20 mM lactate respectively for 48 h. MCT1 expression was quantified by western blot analysis (**A**). Levels of lactate in cell culture supernatants were quantified by colorimetric method (**B**). E-cad, Vimentin and ɑ-SMA expression levels were quantified by western blot (**E**–**H**). MLE-12 were stained with fluorophore-labeled antibodies against Vimentin (Alexa Fluor 488, green) and DAPI dye (blue) and quantified by Fiji software (original magnification × 600, scale bars correspond to 30 µm, (**C**, **D**). Data are expressed as means ± SD, *p < 0.05, **p < 0.01, ***p < 0.001, ****p < 0.0001 vs. control (CON) group; ^##^ p< 0.01, ^###^p < 0.001, ^####^p < 0.0001 vs. Lac_10 mM_ group (ANOVA)

MCT1 is a key protein in cell membrane transporting monocarboxylic acid. Its expression is closely related to the lactate shuttle. In order to investigate the relationship between lactate and EMT in MLE-12 cells, exogenous lactate of 10 mmol per liter (mM) and 20 mM concentrations were added to MLE-12 medium. After culture for 48 h, the expression levels of proteins in cells were detected by western blot. The results showed both moderate level (10 mM) and abundant level (20 mM) of lactate promoted the expression of MCT1 significantly (Fig. 1A). Lactate detection kit was used to assess concentrations of lactate in the supernatants of cell medium. The result showed that the lactate levels were increased in both Lac_10 mM_ and Lac_20 mM_ group, and the lactate level in Lac_20 mM_ group was significantly higher than that in Lac_10 mM_ group (Fig. 1B). Moderate lactate changed expression fold of E-cadherin, Vimentin and α-SMA slightly indicating nonoccurrence of EMT. However, high level of lactate (20 mM) notably promoted expression fold of Vimentin and α-SMA, and decreased the expression of E-cadherin which revealed apparent EMT (Fig. 1E–H). Meanwhile, Vimentin protein was fluorescently labeled and detected by confocal microscopy (Fig. 1D). Fiji software was used to quantify the immunofluorescence and the result consistent with western blot findings (Fig. 1C).

### MCT1 inhibition further enhanced lactate-mediated EMT

**Fig. 2:**
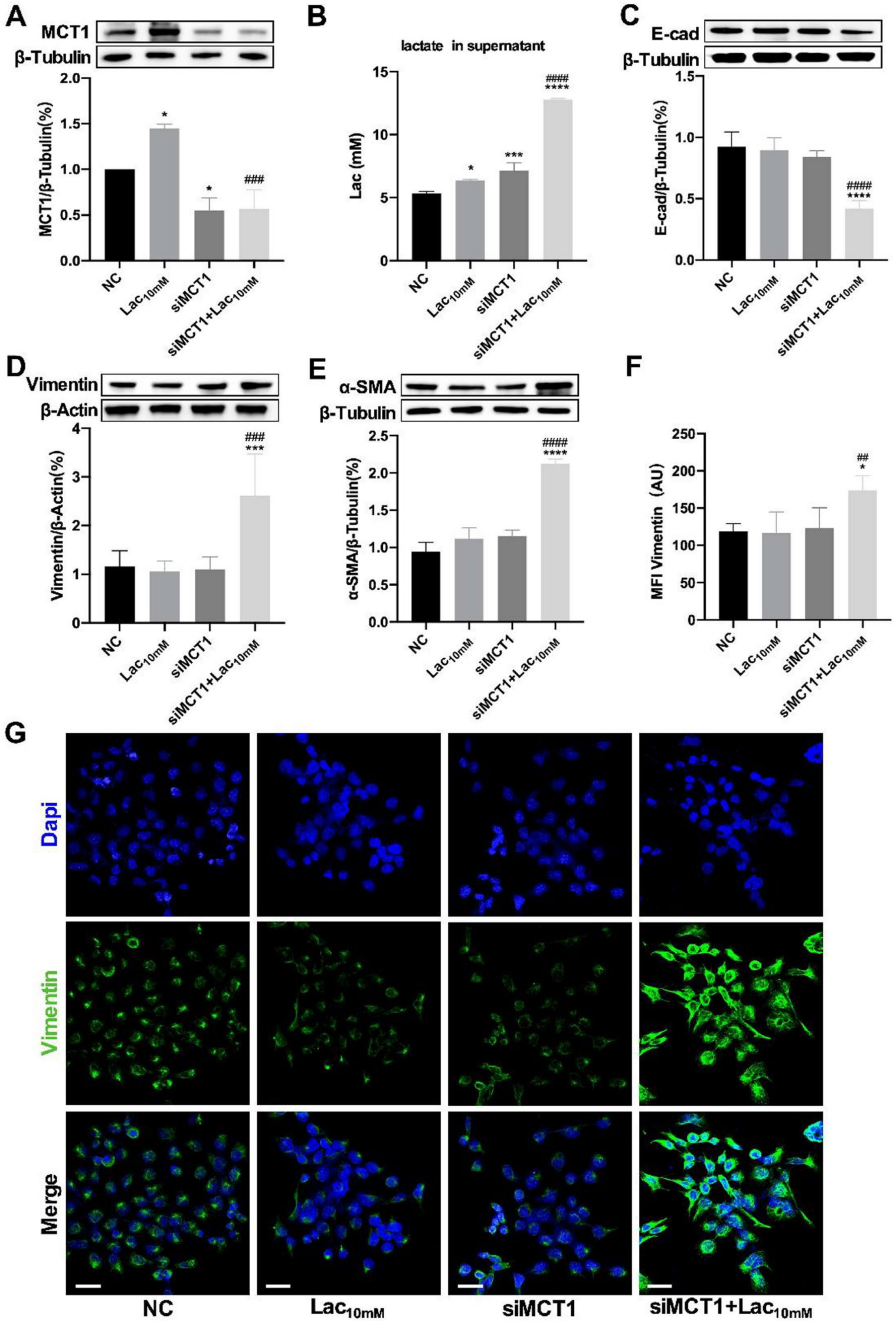
MCT1 inhibition promoted lactate-mediated EMT in alveolar epithelial cells. The expression of MCT1 in MLE-12 cell line was inhibited by siRNA before being subjected to lactate (10 mM). The levels of MCT1 expression were detected by western blot analysis (**A**). Levels of lactate in cell culture supernatants were quantified by colorimetric method (**B**). The expression of EMT marker proteins (E-cadherin, Vimentin, and ɑ-SMA) were measured by western blot (**C**–**E**). MLE-12 cells were stained with fluorophore-labeled antibodies against Vimentin (Alexa Fluor 488, green) and DAPI dye detecting nuclei (blue), and the mean fluorescence intensity were quantified by Fiji software (Original magnification × 600, scale bars correspond to 30 µm, **F**, **G**). Data are expressed as means ± SD, *p < 0.05, ***p < 0.001, ****p<0.0001,  vs. negative control (NC) group; ^##^p< 0.01, ^###^p < 0.001, ^####^p < 0.0001 vs. Lac_10 mM_ group (ANOVA)

To determine the role of MCT1 involved in the lactate-mediated EMT process in alveolar epithelial cells, the expression of MCT1 was knocked down by MCT1-siRNA in MLE-12 cells (Fig. 2A). The results showed that MCT1-siRNA sharply raised concentration of lactate in the MLE-12 culture supernatant after lactate (10 mM) exposure compared with lactate treatment alone (Fig. 2B). The EMT symbols detection showed that the epithelial marker protein E-cadherin was significantly decreased in the siMCT1 + Lac_10 mM_ group (Fig. 2C), while the mesenchymal marker proteins, Vimentin and α-SMA, were significantly increased (Fig. 2D, E). Immunofluorescence results showed Vimentin protein was upregulated apparently in siMCT1 + Lac_10 mM_ group with higher intensity of green fluorescence compared with other groups (Fig. 2F, G). These results indicated that inhibition of MCT1 enhanced EMT process despite with low level of lactate exposure, while EMT was absent in Lac_10 mM_ group. We used the A549 cell line to repeat the experiments and obtained similar results (See Supplementary Fig. 1).

### MCT1 upregulation blocked lactate-induced EMT

**Fig. 3:**
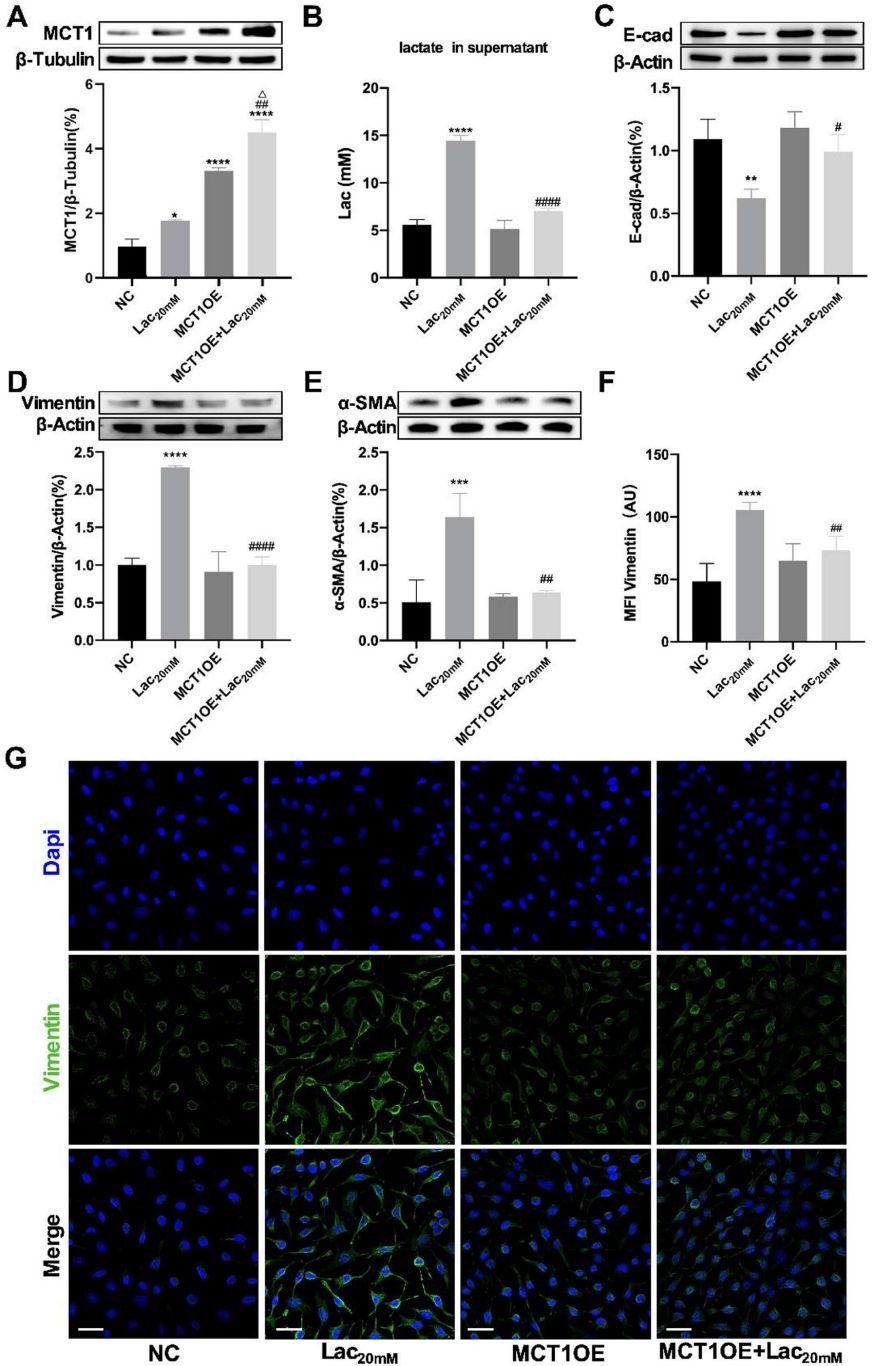
MCT1 upregulation blocked lactate-induced EMT. MLE-12 and MCT1OE cells were treated with PBS (NC and MCT1OE group) or lactate (20 mM) (Lac_20 mM_ and MCT1OE + Lac_20 mM_ group) respectively. MCT1 expression was quantified by western blot analysis (**A**). Levels of lactate in cell culture supernatants were quantified by colorimetric method (**B**). The expression of EMT-related protein (E-cad, Vimentin and ɑ-SMA) was detected by Western blot (**C**–**E**). Cells were stained with fluorophore-labeled antibodies against Vimentin (Alexa Fluor 488, green) and DAPI stain was used to detect nuclei (blue), while the mean fluorescence intensity was quantified by Fiji software (Original magnification × 600, scale bars correspond to 30 µm, **F**, **G**). Data are expressed as means ± SD, *p < 0.05, **p < 0.01, ***p < 0.001, ****p < 0.0001 vs. NC group; ^#^p < 0.05, ^##^p < 0.01, ^####^p < 0.0001 vs. Lac_20 mM_ group; ^△^p < 0.05 vs MCT1OE group (ANOVA)

In order to further explore the role of MCT1 in lactate-induced EMT in MLE-12 cells, MCT1OE lentivirus was transfected with MLE-12 cells to obtain MCT1OE stable cell line. Afterwards, lactate (20 mM) was used to stimulate MLE-12 cells and MCT1OE cells. The protein expression levels of MCT1 were detected by western blot. It was found that MCT1OE significantly improved MCT1 expression, while extra lactate (20 mM) further increased MCT1 level (Fig. 3A). Lactate assay exhibited notably descending level of lactate in cell culture supernatants in MCT1OE + Lac_20 mM_ group compared with Lac_20 mM_ group (Fig. 3B). To verify whether MCT1 overexpression could inhibit EMT process induced by lactate, EMT marker proteins were tested. The results showed that E-cad was significantly reduced in the Lac_20 mM_ group (Fig. 3C), while Vimentin and ɑ-SMA proteins were increased (Fig. 3D, E). Nevertheless, MCT1OE reversed the changes of EMT marker proteins (Fig. 3C–E). Immunofluorescence results also showed that MCT1 overexpression with lactate (20 mM) exposure did not change the fluorescence intensity of Vimentin proteins (Fig. 3F, G).

### LPS promoted EMT in MLE-12 cells through MCT1 inhibition and lactate accumulation

**Fig. 4:**
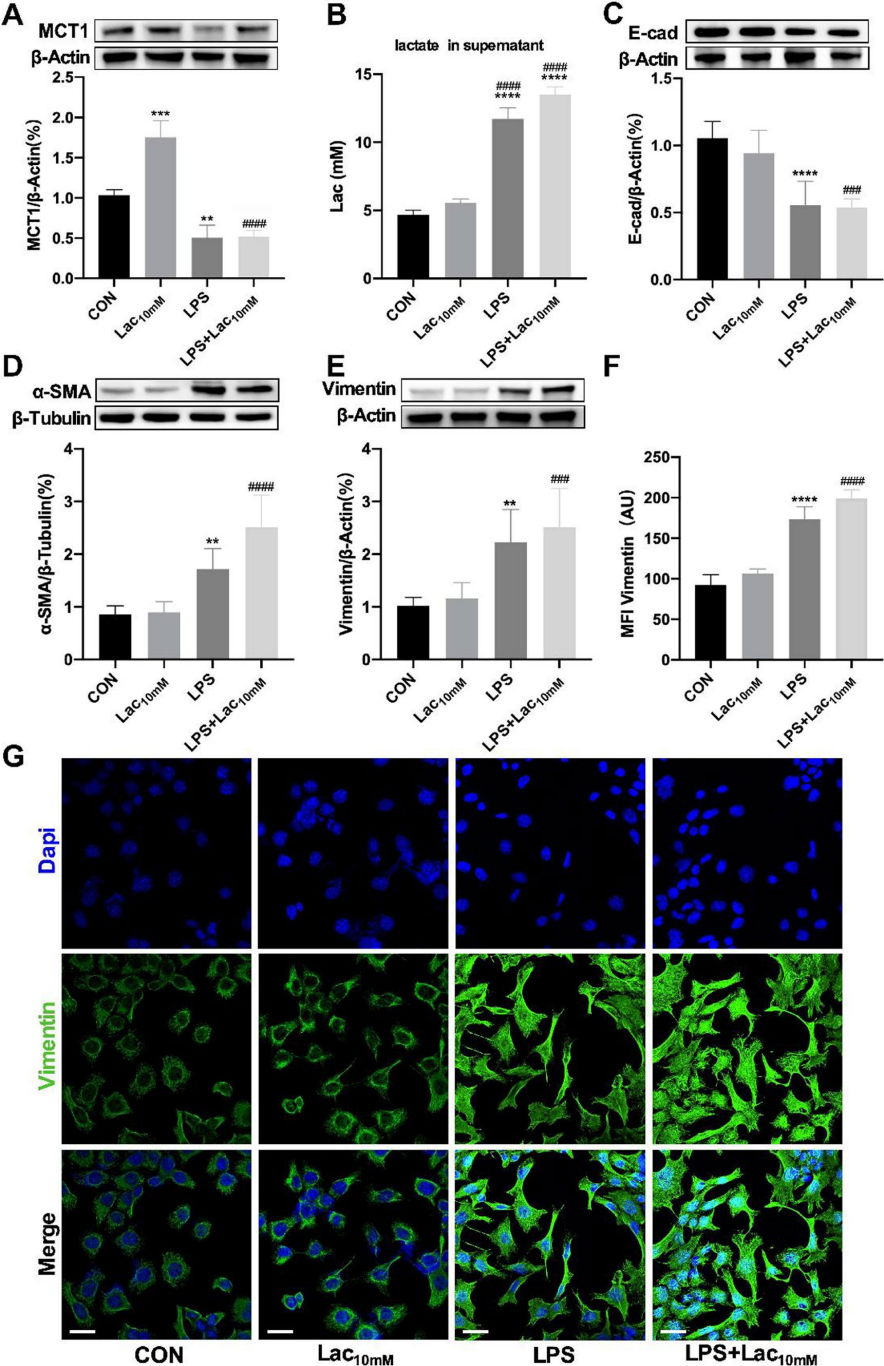
LPS promoted EMT in MLE-12 cells through MCT1 inhibition and lactate accumulation. MLE-12 was divided into four groups: CON group, treated with PBS; Lac_10 mM_ group, treated with lactate (10 mM); LPS group, treated with LPS (1 µg/mL); LPS + Lac_10 mM_ group, treated with LPS (1 µg/mL) and lactate (10 mM). MCT1 expression was quantified by western blot analysis (**A**). Levels of lactate in cell culture supernatant were quantified by colorimetric method (**B**). The expression of EMT-related proteins (E-cad, Vimentin and ɑ-SMA) were detected by Western blot (**C**–**E**). Cells were stained with fluorophore-labeled antibodies against Vimentin (Alexa Fluor 488, green). DAPI stain was used to detect nuclei (blue). The mean fluorescence intensity was quantified by Fiji software (original magnification × 600, scale bars correspond to 30 µm, **F**, **G**). Data are expressed as means ± SD, **p < 0.01, ***p < 0.001, ****p < 0.0001 vs. CON group; ^##^p < 0.01, ^###^p < 0.001, ^####^p < 0.0001 vs. Lac_10 mM_ group (ANOVA)

To investigate cellular effect of LPS on MCT1 expression and lactate metabolism, MLE-12 cells were stimulated by a combination of lactate and LPS for 48 h. Western blot assay showed that the expression of MCT1 in the Lac_10 mM_ group was higher compared with control (CON) group, the expression of MCT1 in the LPS group was lower than CON group, and the expression of MCT1 in the Lac_10 mM_ + LPS group was lower compared with Lac_10 mM_ group (Fig. 4A). In order to examine the change of lactate in the supernatant, the lactate detection kit was applied. The lactate concentrations in the CON group and Lac_10 mM_ group were similar. The level of lactate in the LPS group was significantly higher compared with CON group, and lactate levels in Lac_10 mM_ + LPS group was also higher than Lac_10 mM_ group (Fig. 4B). The data above indicates that LPS could inhibit the expression of MCT1 and cause impaired intracellular transport of lactate and increased level of lactate in the extracellular fluid. Meanwhile, EMT-related marker proteins were detected by western blot. E-cad was decreased in the LPS group and Lac_10 mM_ + LPS group compared with CON group and Lac_10 mM_ group respectively (Fig. 4C). In the contrary, Vimentin and ɑ-SMA proteins were significantly increased in the cells (Fig. 4D, E). The Vimentin protein was also fluorescently detected by antibody labeled with Alexa Fluor 488, observing that Vimentin was significantly enhanced by LPS alone or combination of LPS and lactate (10 mM) (Fig. 4F, G). We used the A549 cell line to repeat the experiments and obtained similar results (See supplementary Fig. 2).

### MCT1 overexpression attenuated LPS-induced EMT in MLE-12 cells

**Fig. 5:**
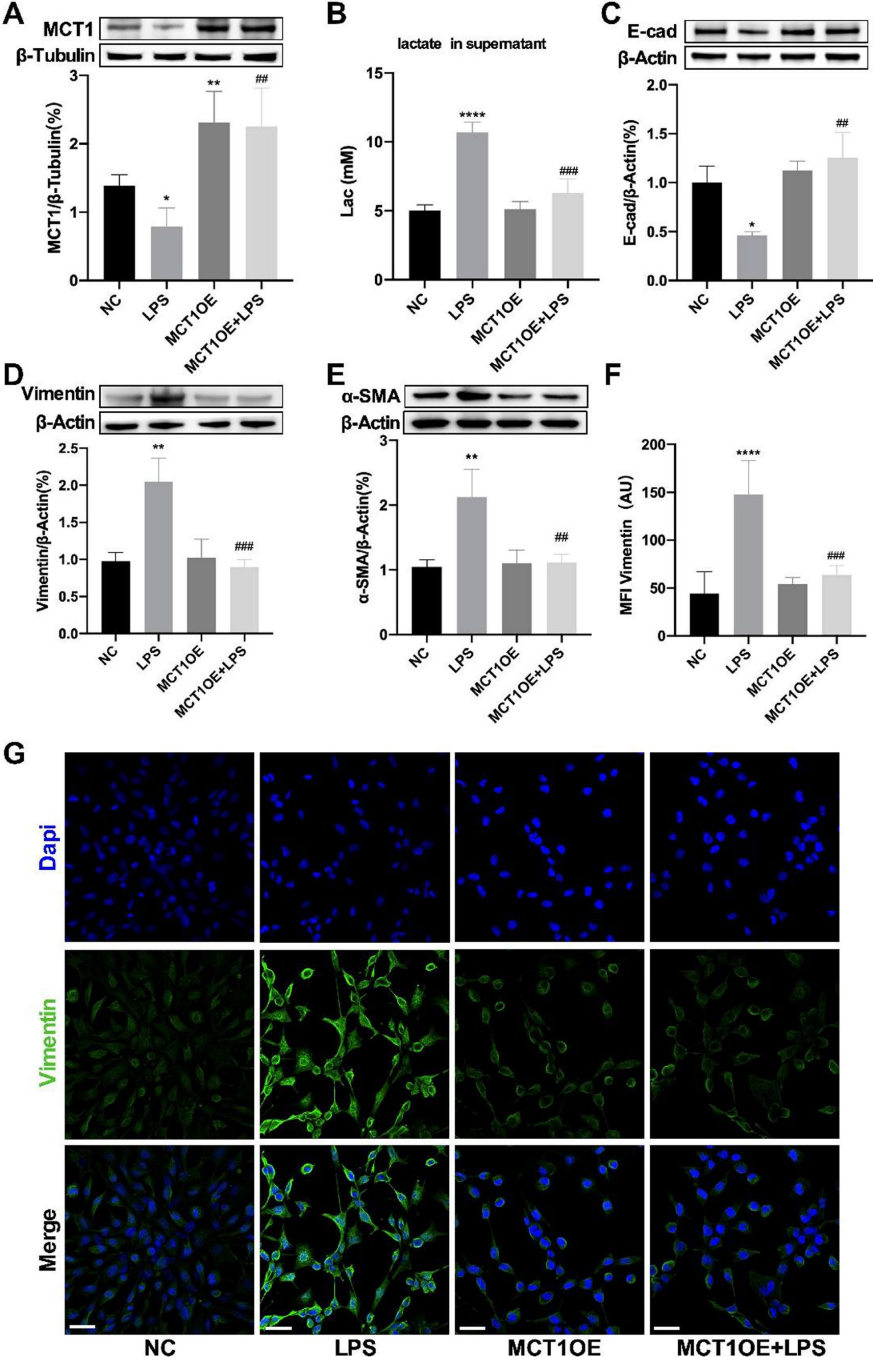
MCT1 overexpression attenuated LPS-induced EMT in MLE-12 cells. MLE-12 and MCT1OE cells were treated with PBS (NC and MCT1OE group) or LPS (1μg/mL) (LPS and MCT1OE + LPS group) respectively. MCT1 expression was quantified by western blot analysis (**A**). Levels of lactate in cell culture supernatant were quantified by colorimetric method (**B**). The expression of EMT-related proteins (E-cad, Vimentin and α-SMA) were detected by western blot (**C**–**E**). Cells were stained with fluorophore-labeled antibodies against Vimentin (green) and DAPI stain was used to detect nuclei (blue). The mean fluorescence intensity was quantified by Fiji software (original magnification × 600, scale bars correspond to 30 µm, **F**, **G**). Data are expressed as means ± SD, **p < 0.01, ***p < 0.001, ****p < 0.0001 vs. NC group; ^##^p < 0.01, ^###^p < 0.001 vs. LPS group (ANOVA)

To further investigate whether LPS led to reduction of MCT1 and disfunction of lactate metabolism and EMT, the MCT1OE cells were subjected to LPS for 48 h. Western blot detection of MCT1 protein showed that LPS-treated cells were observed decreased level of MCT1, while MCT1OE cells overcame LPS injury exhibiting ascending level of MCT1 (Fig. 5A). The lactate concentration in the cell supernatant was detected by colorimetric method, and it was found that the LPS-stimulated MCT1OE cells showed a significant decrease in lactate concentration compared with LPS group (Fig. 5B). According to western blot detection of E-cadherin protein in different groups, we found that the expression of E-cadherin protein in the MCT1OE + LPS group was reversed compared with LPS group (Fig. 5C), as well as Vimentin and ɑ-SMA protein (Fig. 5D, E). The cellular expression of Vimentin protein was detected by immunofluorescence technique, and the results were in consistent with western blot findings (Fig. 5F, G). In brief, it was indicated that increased MCT1 expression could reverse LPS-induced lactate accumulation and EMT process in mouse alveolar epithelial cells.

### LPS diminished MCT1 expression leading to EMT and pulmonary fibrosis

**Fig. 6:**
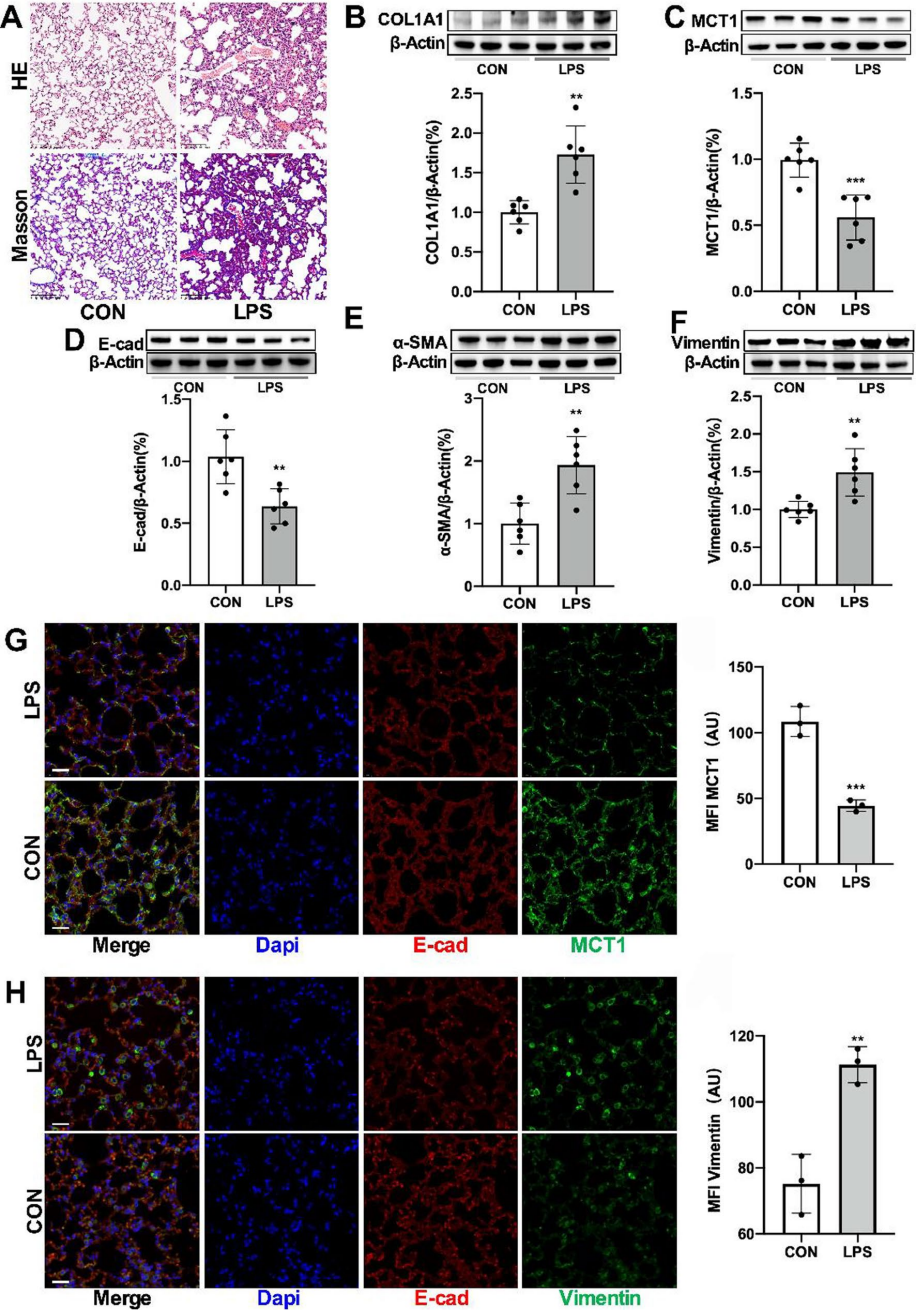
LPS diminished MCT1 expression leading to EMT and pulmonary fibrosis**.** Mice were divided into control and LPS-treated group administrated intraperitoneally with saline or LPS (5 mg/kg/d for 3 consecutive days) respectively. Pulmonary fibrosis was measured by H&E and Masson staining (**A**). Level of COL1A1, MCT1 and EMT relevant markers (E-cad, Vimentin and ɑ-SMA) were quantified by western blot analysis (**B**–**F**). Coexpression of E-cadherin, MCT1 and Vimentin Cells were assessed fluorescently and quantified by Fiji software (**G**, **H**). Original magnification × 600, scale bars correspond to 30 µm. Data are expressed as means ± SD, **p < 0.01, ***p < 0.001 vs. CON group (ANOVA)

A sepsis-associated pulmonary fibrosis mice model was constructed by intraperitoneal injection of LPS (5 mg/kg/d for 3 consecutive days). Samples were collected 7 days after LPS injection. It was found that LPS triggered pulmonary fibrosis obviously according to H&E and Masson staining images (Fig. 6A) and rising level of collagen 1 α1 (COL1A1) protein (Fig. 6B). Besides, the pulmonary level of MCT1 protein was declined in LPS group (Fig. 6C). Meanwhile, LPS was proved to cause pulmonary EMT by western blot detection of EMT marker proteins. E-cadherin was reduced concurrent with increased level of Vimentin and α-SMA (Fig. 6D–F). Similarly, E-cad (red), MCT1 (green) and vimentin (green) proteins were labeled by immunofluorescent antibodies to detect the coexpression of proteins, and the results were consistent with those of western blot (Fig. 6G, H).

### MCT1 overexpression minimized sepsis-associated pulmonary fibrosis

**Fig. 7:**
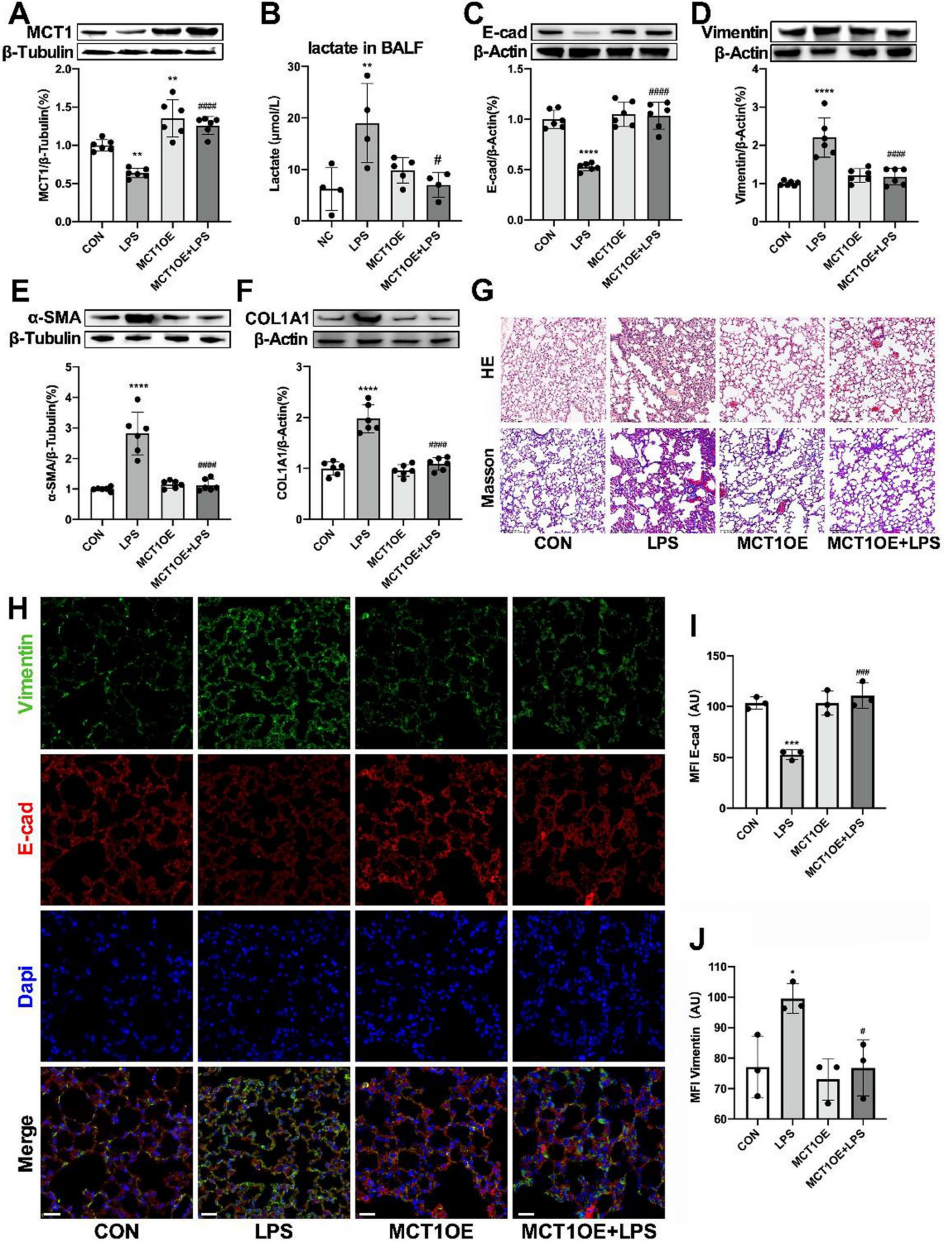
MCT1 overexpression minimized sepsis-associated pulmonary fibrosis. Mice were treated intratracheally with MCT1OE AAV 4 weeks before LPS (5 mg/kg/d) treatment. MCT1 expression was quantified by western blot analysis (**A**). Lactate in bronchoalveolar lavage fluid (BALF) was quantified by ELISA (**B**). The expression of EMT-related proteins (E-cadherin, Vimentin, ɑ-SMA) and COL1A1 were detected by Western blot (**C**–**F**). The severity of pulmonary fibrosis was determined by H&E staining and Masson staining. Original magnification × 300, scale bars correspond to 100 µm (**G**). Lung tissues were stained with fluorophore-labeled antibodies against Vimentin (green), E-cadherin (red) and DAPI stain was used to detect nuclei (blue). Fiji software was used to quantify the immunofluorescence (**I**, **J**). Original magnification × 600, scale bars correspond to 30 µm (**H**). Data are expressed as means ± SD, **p < 0.01, ***p < 0.001, ****p < 0.0001 vs. CON group; ^#^p < 0.05, ^####^p < 0.0001vs. LPS group (ANOVA)

To establish an alveolar epithelial cells specific MCT1OE mouse model, a volume of 50μl MCT1OE AAV mixture (titer: 5e12 vg/ml) was intratracheally administrated 4 weeks before the LPS or saline intraperitoneal injection. As shown in western blot results, MCT1 expression was augmented in either MCT1OE group or MCT1OE + LPS group compared with CON or LPS group (Fig. 7A). In addition, we found that LPS raised lactate concentration in BALF, while overexpression of MCT1 in mice could reverse LPS-induced elevation of lactate level (Fig. 7B). It was also demonstrated that MCT1 overexpression reversed LPS-induced pulmonary EMT evidently according to western blot detection of EMT marker proteins (Fig. 7C–E). Similarly, E-cad (red) and vimentin (green) proteins were labeled by immunofluorescent antibodies to detect the coexpression of proteins, and the results were consistent with those of western blot (Fig. 7H–J). Subsequently, the expression of COL1A1 protein in lung tissues was detected by western blot (Fig. 7F), associated with H&E and Masson staining for pulmonary fibrosis observation. The overexpression of MCT1 in mice could reverse the damage of alveolar structure and collagen deposition caused by LPS (Fig. 7G). In short, these findings indicated that LPS blocked MCT1 expression, led to lactate accumulation, triggered EMT, and consequently generated pulmonary fibrosis in mice. This septic lung damage can be reversed by MCT1 overexpression thus preventing LPS-induced pulmonary fibrosis.

## Discussion

Sepsis is a life-threatening organ dysfunction caused by a dysregulated host response to infection [19], while a sharply rise of serum lactate always indicates deteriorative outcomes. Researchers have found that there is an increased level of aerobic glycolysis and lactate production in fibrotic lung tissue [10, 11, 20], and our previous research also demonstrated that LPS could induce lung fibroblast aerobic glycolysis and lactate production through PI3K-Akt-mTOR/PFKFB3 pathway activation, thus promoting collagen synthesis and pulmonary fibrosis [13]. The role of elevated lactate levels in the process of LPS-induced sepsis-associated pulmonary fibrosis is still unclear. In this study, we demonstrated that during sepsis, LPS inhibits the expression of MCT1, causes abnormal accumulation of lactate in extracellular fluid, thereby initiating the EMT process of alveolar epithelial cells, and finally progresses to pulmonary fibrosis. What’s more, upregulating the expression of MCT1 could restore normal transport of lactate, thus preventing EMT process and pulmonary fibrosis in vitro and in vivo.

EMT is a process during which cells lose their epithelial characteristics and gain mesenchymal properties [21]. Studies have shown that EMT is one of the important sources of lung fibroblasts, which is closely related to the development of pulmonary fibrosis [7, 22, 23]. Some studies have found that the progression of sepsis-associated pulmonary fibrosis is also related to the initiation of EMT, and the EMT process begins in the early stage of the disease [24]. This study has demonstrated that lactate above a certain concentration could trigger the process of EMT in alveolar epithelial cells, thereby promoting pulmonary fibrosis. By stimulating mouse alveolar epithelial cells with varying concentrations of exogenous lactate, we have observed that high concentrations of lactate induced EMT while moderate concentrations did not. In vitro, the up-regulation of MCT1 expression can regulate lactate transport and reduce extracellular lactate concentration, thereby reversing the EMT process of alveolar epithelial cells. Our findings also demonstrate that upregulating MCT1 expression can reverse the EMT process and reduce the degree of lung fibrosis in vivo. This study confirmed that the abnormally elevated lactate level in lung tissue can induce the EMT process of alveolar epithelial cells, which may be an important factor in the occurrence and development of sepsis-associated pulmonary fibrosis.

Lactate is a byproduct of glucose metabolism that can be generated via either aerobic or anaerobic glycolysis [25]. In patients with sepsis and septic shock, hyperlactatemia is frequently observed, and persistent hyperlactatemia and lactic acidosis are strongly associated with mortality in patients with sepsis [26]. In the field of critical care medicine, lactate has traditionally been viewed as a metabolite produced by hypoxic cells undergoing anaerobic glycolysis during septic shock [27]. However, with further research into lactate metabolism, Brooks et al. have proposed the concept of "lactate shuttle", which highlights lactate's crucial role as a carbon source for aerobic energy production and gluconeogenesis throughout the body [28]. Lactate can either be utilized by its producing cells or transported to neighboring or distant tissues and organs. MCT1 is a crucial family of transmembrane transporters found in mammalian cell membranes, responsible for the transportation of monocarboxylate metabolites such as lactate across the membrane [14, 29]. Under normal conditions, MCT1 facilitates lactate influx into cells from extracellular fluid, thereby reducing extracellular lactate concentration [30–32].

MCT1 is also expressed in alveolar epithelial cells, which can transport lactate produced by glycolysis of adjacent cells or distant organs into the cell interior, thereby reducing the local lactate concentration in lung tissue [33]. In this study, in order to clarify the effect of MCT1 on lactate metabolism, siRNA was used to inhibit the expression of MCT1 in MLE-12 cells while MCT1OE cell line was utilized as well. When a moderate dose of exogenous lactate was added to the cell culture medium, MCT1 facilitated its transport into the cells, thus the addition of 10 mM lactate did not result in an increase in its concentration within the supernatant of the Lac_10 mM_ group compared to that of the control group, nor did it induce EMT in alveolar epithelial cells. However, upon inhibition of MCT1 expression via siRNA, there was a significant rise in lactate concentration within the supernatant of siMCT1 + Lac_10 mM_ group as opposed to that observed for Lac_10 mM_ group, which eventually caused EMT of alveolar epithelial cells (refer to Fig. 2). In addition, after adding high concentration of exogenous lactate to the cell culture medium, the lactate concentration in the supernatant of Lac_20 mM_ was significantly higher than that of the control group, which may exceed the transport capacity of MCT1 and lead to EMT in the Lac_20 mM_ group. However, after overexpression of MCT1 expression, the concentration of lactate in the supernatant of the MCT1OE + Lac_20 mM_ group was lower than that of the Lac_20 mM_ group, and the degree of EMT was alleviated (refer to Fig. 3). By regulating the expression of MCT1, the extracellular lactate concentration can be reduced, thereby preventing the EMT process of alveolar epithelial cells.

Our previous study confirmed that LPS leads to increased lactate production during sepsis by activating the key glycolytic enzyme PFKPF3 [13]. Meanwhile, LPS could also inhibit the transport of lactate into the cells by inhibiting the expression of MCT1, leading to further increase of extracellular lactate concentration. As we mentioned before, lactate is a trigger of TGF-β1, extracellular accumulation of lactate can cause a decrease in extracellular pH and activate TGF-β1, EMT process was then initiated, and lung fibrosis subsequently [10]. In vitro, we used LPS combined with moderate dose of lactate to stimulate mouse alveolar epithelial cells. In Lac_10 mM_ group, MCT1 expression was higher than that in the control group, lactate level in the supernatant was not different from that in the control group, and EMT process did not occur; In LPS + Lac_10 mM_ group, MCT1 expression was significantly lower than that in Lac_10 mM_ group, resulting in a significant increase in the concentration of lactate in the supernatant, which further led to EMT of alveolar epithelial cells. In vivo, LPS reduced MCT1 protein in lung tissue, increased lactate concentration in BALF, and caused EMT and tissue fibrosis. Overexpression of MCT1 could significantly reduce the level of BALF lactate, alleviate LPS-induced EMT process, and reverse the process of pulmonary fibrosis.

Our research elucidated that the process of MCT1 transporting lactate could be inhibited by LPS during sepsis, which caused extracellular accumulation of lactate, thus induced EMT in the alveolar epithelial cells and led to pulmonary fibrosis eventually. We creatively identified the important role of lactate and its transporter MCT1 in the EMT process during sepsis-associated pulmonary fibrosis, implying that precise regulation of MCT1 could be a potential therapeutic target in the near future. However, there are also several limitations in this study. Intracellular lactate levels were not examined, which needs to be explored in our future studies. Besides, although our current study demonstrated that lactate accumulation induced by MCT1 inhibition during sepsis did play a role in LPS-induced EMT and pulmonary fibrosis, the role of other monocarcarboxylates cannot be excluded, since MCT1 can also transport other monocarboxylates across the cell membrane as well. Future studies will be carried out to investigate this issue in detail. What’s more, the concrete mechanism of how lactate promoting or LPS inhibiting MCT1 expression during sepsis is still unknown, which warrants further investigation. Based on previous reports, it is hypothesized that lactate or LPS may affect MCT1 expression in alveolar epithelial cells through GPR81- peroxisome proliferator-activated receptor gamma coactivator-1α (PGC-1α) pathway [15, 34–36].

## Conclusion

In summary, this study demonstrates that LPS inhibits the expression of MCT1 causing lactate clearance disorder, leads to lactate accumulation in extracellular fluid and EMT process in alveolar epithelial cells, and ultimately progresses to pulmonary fibrosis. Upregulating MCT1 expression could accelerate lactate intracellular transport and thus attenuate LPS-induced EMT and pulmonary fibrosis.

### Supplementary Information

Below is the link to the electronic supplementary material.

Supplementary file1 (DOCX 9985 KB)

## Data Availability

The datasets generated and analyzed during the current study are available from the corresponding author on reasonable request.

## Abbreviations

AAV: Adeno-associated virus
ARDS: Acute respiratory distress syndrome
AU: Arbitrary units
BALF: Bronchoalveolar lavage fluid
BSA: Bovine serum albumin
COL1A1: Collagen-I α-1
DAPI: 4′6-Diamino-2-phenylindole
EMT: Epithelial-mesenchymal transformation
E-cad: E-cadherin
FBS: Fetal bovine serum
H&E: Hematoxylin and eosin
LPS: Lipopolysaccharide
MCT1: Monocarboxylate transporter-1
MCT1OE: MCT1-overexpressing
MFI: Mean fluorescence intensity
RT: Room temperature
TGF-β1: Transforming growth factor-β1
α-SMA: α-Smooth muscle actin
