# Supplementary material for: LPS-induced monocarboxylate transporter-1 inhibition facilitates lactate accumulation triggering epithelial-mesenchymal transformation and pulmonary fibrosis
Source: Cell Mol Life Sci. 2024 May 6;81(1):206. doi: 10.1007/s00018-024-05242-y (PMC11073230; doi:10.1007/s00018-024-05242-y)
Supplement: Supplementary file 1 — Supplementary file1 (DOCX 9985 KB) [file 18_2024_5242_MOESM1_ESM.docx]

**Supplementary file**

**Supplementary figure 1:**

**MCT1** **inhibition** **further enhanced lactate-mediated EMT in A549 cells**

To investigate the role of MCT1 in lactate-mediated EMT in A549 cells, we utilized AZD3965 (100nM, MedChemExpress, China), a selective inhibitor of MCT1, on A549 cells. Our findings demonstrated that AZD3965 did not affect the expression of MCT1 protein (Supplementary figure 1A-B), but AZD3965 sharply raised concentration of lactate in the A549 culture supernatant after lactate (10mM) exposure compared with lactate treatment alone (Supplementary figure 1C). EMT analysis revealed a significant decrease in the epithelial marker protein E-cadherin in the AZD3965+Lac_10mM_ group (Supplementary figure 1D), while the mesenchymal marker proteins Vimentin, α-SMA and COL1A1 exhibited a significant increase (Supplementary figure 1E, F). Immunofluorescence results indicated a notable up-regulation of Vimentin protein in the AZD3965+Lac_10mM_ group, as evidenced by the higher green fluorescence intensity compared to other groups, and a notable up-regulation of MCT1 protein in the Lac_10mM_ group and AZD3965+Lac_10mM_ group (Supplementary figure 1H). These findings suggest that inhibiting MCT1 activity promotes EMT progression in response to low levels of lactate exposure, while EMT is not observed in the Lac_10mM_ group.


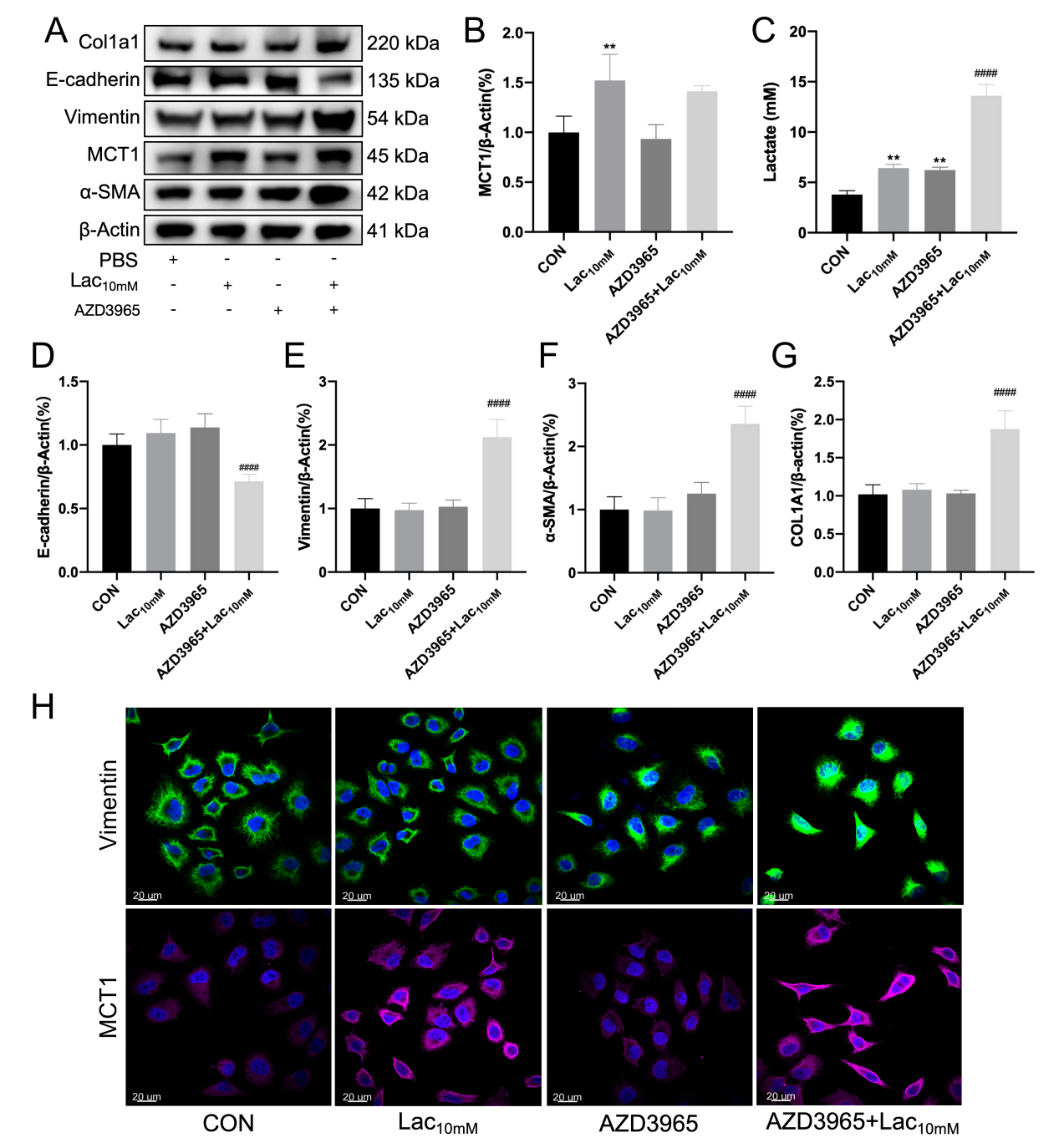


**Supplementary figure 1. MCT1 inhibition promoted lactate-mediated EMT in** **A549 cells.**

The levels of MCT1 expression were detected by western blot analysis (B). Levels of lactate in cell culture supernatants were quantified by ELISA (C). The expression of EMT marker proteins (E-cadherin, Vimentin and ɑ-SMA) and COL1A1 were measured by western blot (D-G). A549 cells were stained with fluorophore-labeled antibodies against Vimentin (Alexa Fluor 488, green), MCT1((Alexa Fluor 488, purple red) and DAPI dye detecting nuclei (blue). Original magnification × 600, scale bars correspond to 20µm. Data are expressed as means ± SD, ^**^p<0.01, vs. CON group; ^####^p<0.0001 vs. Lac_10mM_ group (ANOVA).

**Supplementary figure 2:**

**LPS promoted EMT in A549 cells through MCT1 inhibition and lactate accumulation**

A549 cells were used to observe the effect of LPS on cellular MCT1 expression and lactate metabolism. Lactate and LPS were combined to stimulate A549 cells for 48 hours. The expression of related proteins in cells were detected by Western blot. We found that the expression of MCT1 was higher in the Lac_10mM_ group than in the CON group, lower in the LPS group than in the CON group, and lower in the Lac_10mM_+LPS group than in the Lac_10mM_ group (Supplementary figure 2B). The content of lactate in the supernatant was detected by ELISA. Lactate concentrations were similar in CON and Lac_10mM_ groups. Lactate levels in the LPS group were significantly higher than in the CON group and were also higher in the Lac_10mM_+LPS group than in the Lac_10mM_ group (Supplementary figure 2C). The above data suggest that LPS inhibits MCT1 expression, leading to impaired intracellular lactate transport and increased extracellular fluid lactate levels. Western blot was used to detect EMT-related marker proteins. E-cadherin protein expression was decreased in both LPS group and Lac_10mM_+LPS group compared with CON group and Lac_10mM_ group (Supplementary figure 2D). In contrast, Vimentin, ɑ-SMA and COL1A1 were significantly increased in cells (Supplementary figure 2E-G). Fluorescence of MCT1 protein (purple red) and Vimentin protein(green) were stained with Alexa Fluor 488-labeled antibodies, observing that Lactate (10mM) stimulation enhanced MCT1 protein expression compared with CON group, and LPS alone or in combination with lactate (10mM) significantly enhanced Vimentin protein expression (Supplementary figure 2H).


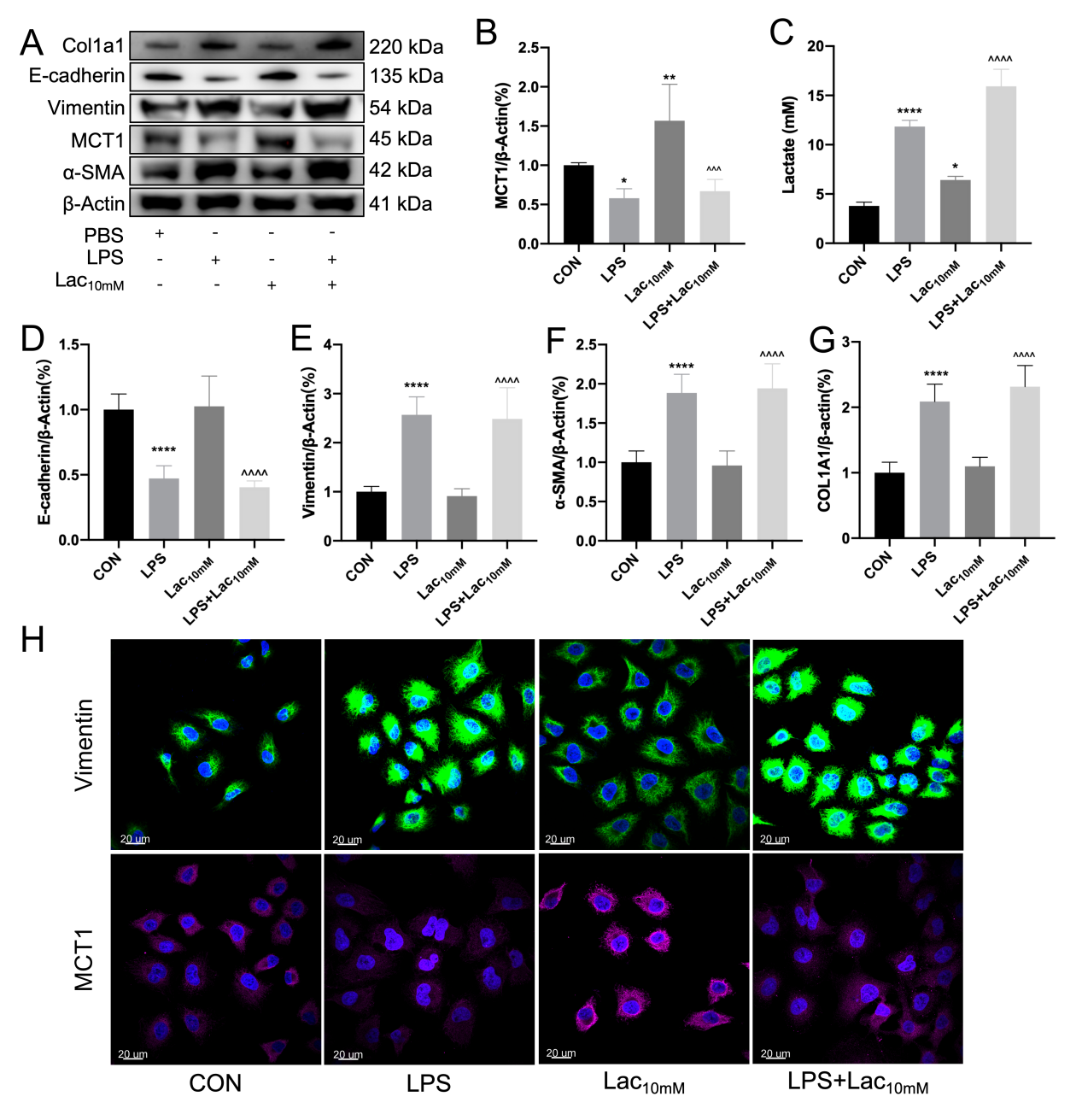


**Supplementary figure 2. LPS promoted EMT in A549 cells through MCT1 inhibition and lactate accumulation.**

A549 cells were divided into four groups: CON group, treated with PBS; LPS group, treated with LPS (1ug/ml); Lac_10mM_ group, treated with lactate (10mM); LPS+Lac_10mM_ group, treated with LPS(1ug/ml) and lactate (10mM). MCT1 expression was quantified by western blot analysis (B). Levels of lactate in cell culture supernatant were quantified by ELISA(C). The expression of EMT-related proteins (E-cadherin, Vimentin and ɑ-SMA) and COL1A1 were detected by Western blot (D-G). Cells were stained with fluorophore-labeled antibodies against Vimentin (Alexa Fluor 488, green) and MCT1(Alexa Fluor 488, purple red). DAPI stain was used to detect nuclei (blue). Original magnification×600, scale bars correspond to 20µm(H). Data are expressed as means±SD, ^*^p<0.05, ^**^p<0.01, ^****^p<0.0001vs. CON group; ^^^^^p<0.001, ^^^^^^p<0.0001vs. Lac_10mM_ group (ANOVA).
